# Non-invasive assessment of leaf water status using a dual-mode microwave resonator

**DOI:** 10.1186/s13007-015-0054-x

**Published:** 2015-02-22

**Authors:** Said Dadshani, Andriy Kurakin, Shukhrat Amanov, Benedikt Hein, Heinz Rongen, Steve Cranstone, Ulrich Blievernicht, Elmar Menzel, Jens Léon, Norbert Klein, Agim Ballvora

**Affiliations:** INRES-Plant Breeding, University of Bonn, Katzenburgweg 5, 53115 Bonn, Germany; EMISENS GmbH, Zur Rur 25, 52428 Juelich, Germany; Department of Materials, Imperial College London, South Kensington Campus, London, SW7 2AZ UK; Dr.- Ing. Elmar Menzel Ingenieurbüro, Birkenstr. 18, 63533 Mainhausen, Germany

**Keywords:** Water content, Microwave resonator, Non-invasive measurements

## Abstract

The water status in plant leaves is a good indicator for the water status in the whole plant revealing stress if the water supply is reduced. The analysis of dynamic aspects of water availability in plant tissues provides useful information for the understanding of the mechanistic basis of drought stress tolerance, which may lead to improved plant breeding and management practices. The determination of the water content in plant tissues during plant development has been a challenge and is currently feasible based on destructive analysis only. We present here the application of a non-invasive quantitative method to determine the volumetric water content of leaves and the ionic conductivity of the leaf juice from non-invasive microwave measurements at two different frequencies by one sensor device. A semi-open microwave cavity loaded with a ceramic dielectric resonator and a metallic lumped-element capacitor- and inductor structure was employed for non-invasive microwave measurements at 150 MHz and 2.4 Gigahertz on potato, maize, canola and wheat leaves. Three leaves detached from each plant were chosen, representing three developmental stages being representative for tissue of various age. Clear correlations between the leaf- induced resonance frequency shifts and changes of the inverse resonator quality factor at 2.4 GHz to the gravimetrically determined drying status of the leaves were found. Moreover, the ionic conductivity of Maize leaves, as determined from the ratio of the inverse quality factor and frequency shift at 150 MHz by use of cavity perturbation theory, was found to be in good agreement with direct measurements on plant juice. In conjunction with a compact battery- powered circuit board- microwave electronic module and a user-friendly software interface, this method enables rapid in-vivo water amount assessment of plants by a handheld device for potential use in the field.

## Background

Drought and salinity stress are undoubtedly important constraints limiting agricultural productivity which can even result in total yield loss [1,2]. To equilibrate the decrease of the uptake of the available water in soils, plants preserve the osmotic potential by reducing stomata conductance. This leads to a reduction of photosynthetic rate and finally reducing plant growth and yield [3,4]. Around 26% of arable land worldwide is suffering from water shortage constituting the most important abiotic stress [5]. In perspective to climate changes in the future an increase of drought stress and consequently problems with plant production [4,6-8] are expected. Understanding the mechanism of drought stress tolerance is in the focus of current plant research, in order to help breeders developing new cultivars that perform well, even under water scarcity.

The definition of the water status in plant tissue is of importance for the plant researcher to better understand the physiological processes and molecular mechanisms leading to tolerance with respect to water lack stress on the one hand. On the other hand it may help the producers to control the watering procedures. Systematic phenotyping of plants needs standardized and non-invasive methods to define and assess physiological parameters like water status in order to analyze the reactions of single plants or group of plants to environmental.

The water content in vegetative tissues is a parameter of high importance for the photosynthetic performance and an indicator of the plant’s health. Currently it is measured by destructive methods such as comparing the fresh and dry weight of plant tissues [9]. Nevertheless, destructive methods do not allow the instantaneous and continuous monitoring of the water content in living tissue. Therefore, non-destructive techniques that require very weak interaction with the plant tissue in order to avoid altering its physiological activities are highly desired.

Non-destructive analysis by radiation in the microwave to terahertz range is most promising for the development of non-invasive methods to determine the water content because of the strong water absorption in this frequency range [10-12]. The selection of frequency is determined by the size of the assessed objects in comparison to the wavelength, if standard absorption or reflection methods are being used. In the case of plant leaves of centimeter dimension, frequencies above about 30 GHz (wavelength λ = 1 cm) are advantageous, in particular the THz range with λ below one millimeter.

Recently, THz measurements have been used to measure the water content in leaves [9,13]. However, THz technology is still quite expensive in comparison to the microwave bands below 20 GHz. Our work represents the first systematic study on individual plant leaves by a dielectric resonator based method, similar to the one described by Menzel, et al. [10], which was developed with direct involvement of one of the authors. Other than in the method described by Menzel, et al. [10], the additional use of a low frequency mode being excited in the same cavity at 150 MHz enables independent and simultaneous non-invasive determination of the ionic conductivity [14]. Different to microwave moisture sensors based on planar microwave transmission lines like the one reported by Rezaei, et al. [15] and planar antennae approaches by Sancho-Knapik, et al. [16] our method allows the determination of the real and imaginary components of the complex dielectric permittivity at two well separated frequencies. Moreover, our evanescent field approach overcomes the wavelength limitation and enables the use of much lower frequencies at 150 MHz and 2.4 GHz, with the advantage of cheap electronic components as being used in wireless communication. The potential commercial availability of an evanescent field dual mode microwave sensor system at moderate cost enables the implementation of non-invasive water and conductivity assessment in biological research laboratories.

### Microwave properties of plant tissue

The microwave properties of plant tissue strongly correlate to the amount of stored water. The typical water content in healthy plant leaves is around 90% [17].

The interaction of microwaves with water, which is determined by a broad absorption peak due to Debye-type molecular relaxation, centered at around 20 GHz at room temperature, can be described by a strongly frequency dependent complex-valued dielectric permittivity,

1 $$ \varepsilon *\left(\omega \right)=\varepsilon \hbox{'}\left(\omega \right)+j\varepsilon \hbox{'}\hbox{'}\left(\omega \right)={\varepsilon}_{\infty }+\frac{\varepsilon_s-{\varepsilon}_{\infty }}{1+{\omega}^2{\tau}^2}+j\left[\frac{\left({\varepsilon}_s-{\varepsilon}_{\infty}\right)\omega \tau }{1+{\omega}^2{\tau}^2}+\frac{\sigma }{\omega {\varepsilon}_0}\right], $$

with *ε*_s_ representing the static dielectric permittivity, *ε*_∞_ the permittivity at *f* → ∞, *τ* the dipole relaxation time of the water molecules and *σ* the ionic conductivity due to dissolved salts or other ions and metabolites [18]. In Eq. 1, the frequency *f* is expressed by the angular frequency *ω* = 2*πf*, *ε*_0_ = 8.85⋅10^−12^ F/m is the vacuum permittivity.

The dielectric properties of liquid water can be well described by Eq. 1 up to about 60 GHz, using temperature dependent values of *ε*_s_, *τ* and *σ* [18,19]. At room temperature (*T* = 22°C), experimental data for distilled water can be well fitted using *ε*_s_ = 78.36, *τ* = 8.27 ps, *ε*_∞_ = 5.16 and *σ =*0 [20]. At 2.4 GHz and 150 MHz, where the experiments are conducted, ε*(2.4 GHz) = 77 + j 9.0 and ε*(150 MHz.2) = 78 + j 0.57, respectively. In particular at 150 MHz, a large contribution of to the conductivity term (3^rd^ term in Eq. 1) by dissolved ions to the imaginary part of ε* can be expected: broadband microwave dielectric measurement on fluids extracted from wheat leaves revealed equivalent NaCl concentrations of around 1% [21], which results in a conductivity of about 17,600 μS/cm, the corresponding imaginary part of ε* at 2.4 GHz and 150 MHz are 13 and 211, respectively (3^rd^ term in Eq. 1). Hence, the ratio Im (ε*_ions_)/Im (ε*_dipole_), which describes the ratio of ionic to dipole losses, comes out to be 1.47 at 2.4 GHz and 370 at 150 MHz for the given conductivity. Therefore, the mode at 150 MHz is ideally suited for non-invasive and contact-free conductivity measurements.

It is worth to note that the Debye relaxation parameters and the ionic conductivity are strongly temperature dependent, therefore it is important that the measurements are performed within well-defined temperature intervall. The dielectric response of the leaf can be understood as an effective medium composed of water with ions and of dry bulk material. In contrast to water, the bulk material has a relatively low permittivity ε ‘ ≤10, and the imaginary part is negligible, as demonstrated by measurements on totally dried leaves (see section about results and discussion) . Therefore, as long as the absolute water content is more than about 10% the contribution of the bulk plant material to the real part of the, dielectric permittivity can be neglected as well. However, as discussed in Ulaby, et al. [21], the calculation of complex permittivity of a representative effective medium would require detailed information about the water distribution within the veins and as inter- and intracellular liquid, because of unequal amounts of water in different tissue compartments. Nevertheless, by assessing dielectric properties of two materials as reported by Sancho-Knapik, et al. [16], a very good correlation between RWC (relative water content) and reflectance at a frequency of 1730 MHz was found both for filter paper and leaves. Therefore, the integral complex permittivity, as determined by microwave dielectric measurement, represents a reasonable experimental quantity which is representative for the water content (or conductivity in case of the imaginary component at 150 MHz) of a leaf under investigation.

According to a comprehensive study within the framework of effective medium theories as described in Ulaby, et al. [21] the static permittivity for fresh wheat leaves is about 35, corresponding to a volumetric moisture of about 60%. This correlation depends on the density of the fresh leaf material, which may vary for different species, but was not analyzed within this study.

## Results and discussion

### The dual mode cavity as leaf sensor

The patented dual mode cavity sensor, which is discussed in detail in Klein, et al. [14], enables simultaneous dielectric measurements at two distinct and far separated frequencies: For the sensor which was employed in this study, one resonant frequency is at 150 MHz (Mode 0), the second one 2.4 GHz (Mode 1). For the study of the correlation between drying status and permittivity we employed Mode 1 only because of large signal-to-noise ratio, i.e. larger frequency shifts in comparison the resonant halfwidth. In spite of poor signal-to-noise ratio, preliminary data by Mode 0 on fresh wheat leaves are discussed. It is worth to note that Mode 0 is ideally suited for contact-free assessment of the ionic conductivity of bulky plant tissues such as potatoes and sugar beets, where the sample volume and hence the signal-to-noise ratio is much larger.

**Figure 1:**
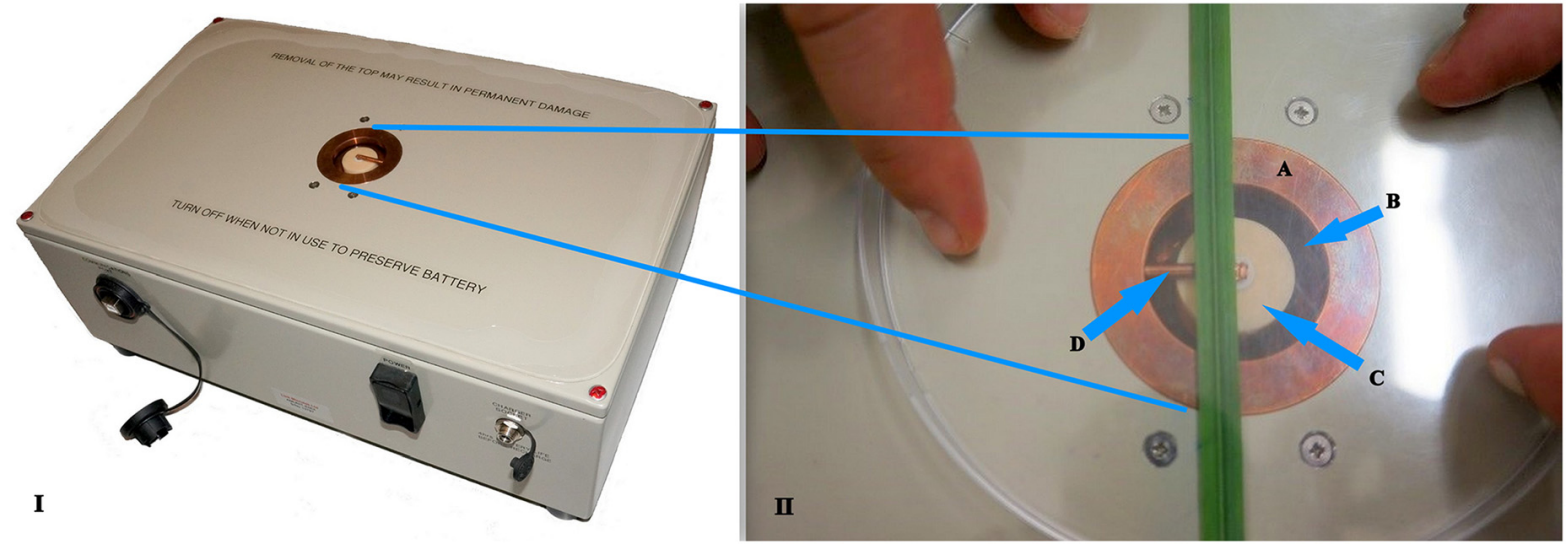
**Microwave sensor.** (I) – Photograph of the employed sensor system comprising a compact battery - powered circuit board - microwave electronic module, and (II) the zoomed measurement-window: dual mode cavity (copper, **A**) embedded in a housing with a wheat leaf in measurement position. The aperture in the copper cavity (dark circle, **B**) allows the evanescent field of the ceramic dielectric resonator (smaller light circle, **C**) to penetrate into the sample under test. The radial copper rod (**D**) which is partially covered by the leaf is a requirement for Mode 0 only.

Mode 1 corresponds to the TE_01δ_-mode [22] of the cylindrically shaped dielectric resonator, embedded in the dual-mode cavity. The evanescent electric field is presented by concentric circles, the field magnitude increases from zero in the center of the aperture towards its maximum at about 2/3 of the radius of the dielectric resonator (light circle in Figure 1,C), and gradually decreases to zero towards the aperture. From the aperture plane (leaf measurement position), the evanescent field decreases exponentially in axial direction and reaches 50% of its value at the top edge of the aperture at a distance of about 20 mm above the aperture. The evanescent field of the lumped element mode (Mode 0) is strongly concentrated in close vicinity of the radial metallic rod, in particular near the center of the cavity [14].

As it will be discussed along with the experimental data, for Mode 1 the magnitude of the leaf induced alteration of the resonant properties depends on the degree of coverage of the aperture by the leaf under test. In case of a partial coverage, as indicated by the wheat leaf shown in Figure 1(II), a strict protocol how to arrange the leaf on the sensor surface is required for each given type of leaf. A smaller aperture would be tempting for the assessment of smaller leaves, but would cause a strong reduction of the electric field amplitude at the leaf position, which leads to a significant reduction of sensitivity.

During the assessment of a leaf under test, the change of the inverse quality factor *Q* and the resonant frequency, *f*_r_ with respect to the empty resonator is recorded. Both *Q* and *f*_r_ are determined from a fit of a Lorentzian to the measured transmission curve using.

2 $$ U(f)=\frac{U\left({f}_r\right)}{\sqrt{1+4{Q}^2{\left(\frac{f}{f_r}-1\right)}^2}} $$

In Eq. 2*U*(*f*) represents the frequency dependent detector voltage, which is proportional to the power transmitted through the resonator (square law detection) upon sweeping the generator frequency around the resonance frequency *f*_r_. Both modes are excited by a different pair of coaxial probes for each, the signals are generated and recorded by two independent electronic modules. Each of the two PCB (printed circuit-board) - based integrated electronic modules is composed of a digitally controlled synthesizer- PLL (phase locked loop) controlled microwave VCO (voltage controlled oscillator) and a detector unit.

Prior to each measurement with a leaf in place, *f*_r_ and *Q* are recorded for the empty resonator. For the analysis, the negative relative frequency shift due to the sample,

3a $$ FRS\equiv -\frac{f_{r, sample}-{f}_{r, empty}}{f_{r, empty}} $$

and the sample induced change of the losses, i.e. change of the inverse Q factor, *IQS*,

3b $$ IQS\equiv \frac{1}{Q_{sample}}-\frac{1}{Q_{empty}} $$

are recorded. Since the frequency shift due to a dielectric object is usually negative, *FRS* is defined to be a positive number. It is important to note that IQS is independent of coupling losses, because coupling leads to a constant 1/*Q* contribution which does not change due the sample in measurement position.

For the case, that the field distribution of the evanescent field is not distorted by the sample, *FRS* and *IQS* can be directly related to the complex permittivity of the sample by extended cavity perturbation theory [23].

4 $$ \begin{array}{l}FRS=\frac{\kappa }{2}\left(\varepsilon \hbox{'}-1\right)\kern9.75em IQS=\kappa \varepsilon \hbox{'}\hbox{'}\\ {}\kappa =\frac{\varepsilon_0{\displaystyle \underset{V}{\int }{E_0}^2dV}}{2W}\end{array} $$

In Eq. 4, the filling factor *κ* describes the electric resonant field energy within the sample of volume, the integral in the numerator extends over the volume fraction *V* of the sample which is exposed to the unperturbed resonator field *E*_*0*_, normalized to the total electric field energy, *W*, of the cavity.

In order to test the applicability of the perturbation approach, electromagnetic field simulations of the cavity-leaf system have been performed with CST Microwave Studio [24] for a variety of configurations. The results indicate that the alteration of the magnitude of the electric field at the position of the leaf due to leaf itself is less than 10% in the worst case assuming a homogenous water distribution inside the leaf. Therefore, the analysis by Eq. 4 is justified within the experimental errors. However, we cannot rule out that water being concentrated in veins may lead to some level redistribution of the local electromagnetic field, which is subject of an ongoing study.

The accurate calculation of the filling factor *κ* requires a detailed analysis of the shape of the leaf and its exact measurement position - along with the electric field distribution of the resonant mode. However, relative measurements of *FRS* and *IQS* for a given leaf in a reproducible measurement position allow the monitoring of relative changes of the complex permittivity. It is worth to mention that the ratio of *IQS* and *FRS* is independent of *κ,* and may represent a size and position independent figure of merit for a given leaf. For Mode 1, even in case of a complete coverage of the aperture, the leaf-induced alteration of resonance frequency and *Q* factor may depend on the exact measurement position of the leaf under test, because the water distribution in the leaves is inhomogeneous. This means, that a maximum of *FRS* and *IQS* is usually achieved if water filled veins are located around the position of maximum field. For the sake of a maximum signal-to-noise ratio, the position was optimized for maximum *FRS*. In case of elongated leaves like wheat the leaf axis was arranged at an offset of about 50–80% of the radius of the dielectric resonator, corresponding to a field maximum of the TE_01d_ mode (Mode 1). The optimization of the position with regards to Mode 0 is subject to a separate analysis and will not be further addressed in this contribution.

However, as indicated in the section about results and discussion, the leaf-induced alterations can be used for a preliminary analysis.

Although the leaf under test is physically attached to the metallic aperture of the cavity in order to ensure a reproducible measurement position, the measurement is contact-less in nature. A thin plastic foil between aperture and sample would not have any significant effect on the results, because the electric field is coupled to the sample inductively, without any need of an electrical contact.

### Measurement of water content in leaves of different plants

The four plant species being analyzed, wheat, maize, potato and canola were selected considering the size and morphology of their leaves. Wheat and maize leaves have similar shape, both are long but wheat leaves are thinner. On the other hand, the potato and canola have compound leaves with oval leaflets, the canola leaves are larger and thicker.

The three leaves detached from each plant were chosen from three developmental stages in order to characterize tissues of various ages. Shortly after removal from the plant, the leave under test was weighted and subsequently measured with the microwave sensor system. The leave was placed on the window such the measured frequency shift is maximized, as shown in Figure 1 for wheat. This first assessment was representative for the fresh leaf and which was considered as reference of 100% (w/w) water content. In fact, the time interval between removal and measurement was less than 30 seconds in any case.

**Figure 2:**
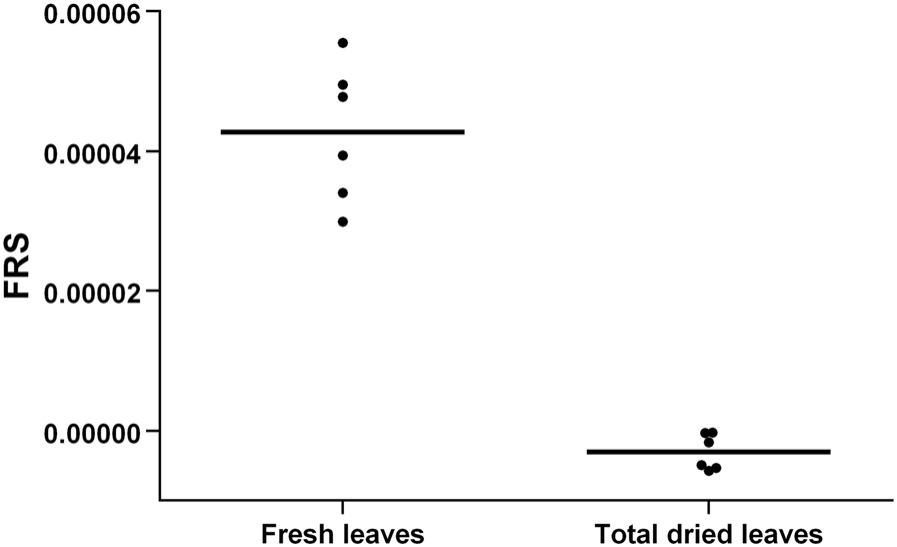
**Comparative dielectric conductivity of fresh and totally dried wheat leaves.** Six leaves in total were measured fresh and subsequently completely dried. The dots represent the average values of five measurements (technical replicates) and the lines the average values of six leaves.

A significant change in the resonant frequency shift of Mode 1 (2.4 GHz) between fresh and dry leaves was demonstrated, which is far beyond the variation from leaf to leaf for a given plant (Figure 2). It is notable that the frequency shift of the dry leaf is zero within the measurement accuracy limits which indicates that water represents the dominant part of the response.

For Mode 0, only wheat leaves have been investigated till date. The measured values of *FRS* and *IQS* are of the same order of magnitude as for Mode 1, but the signal-to-noise ratio is nearly ten times lower than for Mode 1. This is due to the smaller resonant halfwidth of the unloaded resonance, usually expressed by the quality factor *Q*_empty_ without sample, *Q*_empty_(Mode 0) = 350, *Q*_empty_(Mode 1) = 4200).

All measurements where performed at room temperature without any room temperature control. Test measurements on canola and wheat leaves at 18°C, 22°C and 27°C showed no significant differences of the *FRS* or *IQS* values.

**Figure 3:**
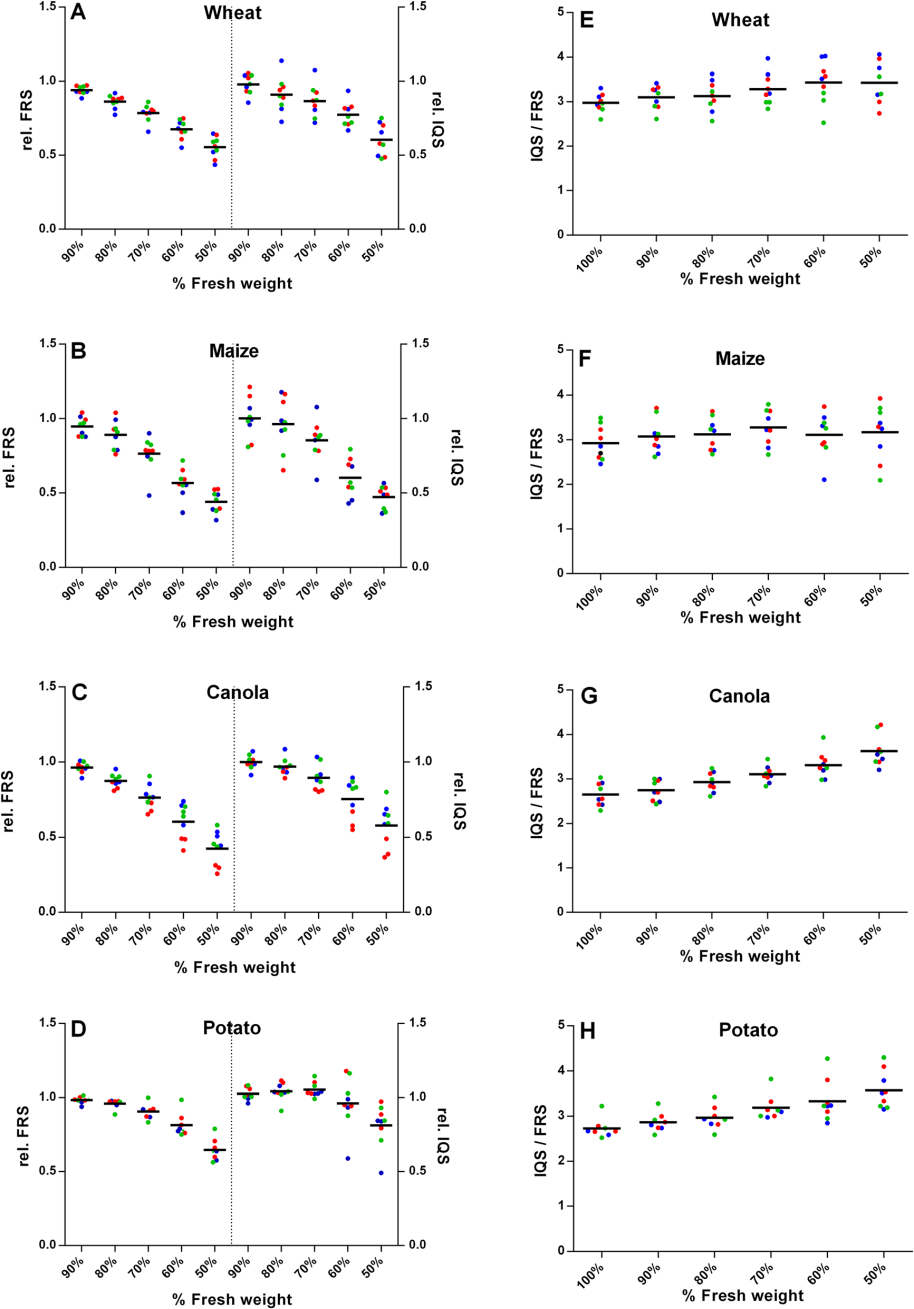
**Experimental results of microwave measurements on leaves from four different crops.** The analysis was performed at six time-points from initial fresh weight (100%) up to 50% of the initial weight by consecutive 10% drying in each step. **(A)** - Wheat, **(B)**-Maize, **(C)** - Canola, and **(D)** - Potato. Normalized FRS and IQS values based on values at 100% fresh weight for FRS and IQS, respectively are shown. **E** (Wheat), **F** (Maize), **G** (Canola) and **H** (Potato ) display IQS values divided by FRS values for single leaves at different drying stages, and water contents, respectively. Bars indicate arithmetic means over three leaves. The absolute averaged FRS values at 100% fresh weight are 5.5⋅10^−5^ for wheat, 1.25⋅10^−4^ for maize, 1.41⋅10^−4^ for canola and 1.24⋅10^−4^ for potato. The colors of the dots indicate the developmental stage of the leaves: blue (first stage, young leaf), green (second stage, intermediate leaf) and red dots (third stage, older leaf).

For Mode 1, the measured values of *FRS* and *IQS* as a function of percentage of fresh weight for four different types of plants were normalized to the average value of the fresh leave (Figure 3). Although the absolute values of *FRS* and *IQS* differ from leaf to leaf due to a different filling factor *κ* (Eq. 4) the normalized *FRS* and *IQS* values exhibit a systematic decrease with increasing weight loss, which indicates that water provides the most significant contribution to the dielectric permittivity of leaf tissue. The data points of individual leaves indicate this trend. The averaged values displayed in Figure 3 as horizontal lines are representative mean values for all leave stages. Based on t-test, significant differences between the mean values were revealed (p < 0.05). Measurement of three leaves and calculation of their mean values show stronger correlations to the water content than the single leaves measurement. Assuming that the mass density of the dry leave tissue is small in comparison to that of water, the weight percentage represents the volumetric water concentration – multiplied by factor of about 0.5-0.7 (maximum water volume concentration in a fresh leaf) [21]. According to our data, the normalized *FRS* values drop to about 0.45-0.55 for wheat and maize, and to slightly higher values of about 0.65-0.75 for potato and canola - as result of weight reduction or water loss from 100% to 50%. It is remarkable, however, that the *FRS* – weight dependences exhibit a recognizable positive curvature and deviate from linearity, in contrast to the slight negative curvature being observed by broadband dielectric measurements on wheat leaves and stalks [21]. For potato leaves and in particular for canola leaves, where a considerable portion of water is stored in relatively thick veins, this effect is most pronounced. We presume that the drying process by evaporation works slower for large veins. As a result, the measured weight may not be representative for the real water concentration in the largest veins: if the largest veins are located close to a field maximum of the resonant field, the measured *FRS* values may overestimate the average water concentration in the leaf, which explains the observed curvature qualitatively.

For the fresh weight data, the average ratio IQS/FRS varies only slightly between 2.8 and 3 for the three different plants. Assuming the validity of the perturbation approach according to Eq. 4, *IQS*/*FRS* is equal to two times the loss tangent.

5 $$ \frac{IQS}{FRS}=\frac{2\varepsilon \hbox{'}\hbox{'}}{\varepsilon \hbox{'}-1}\approx \frac{2\varepsilon \hbox{'}\hbox{'}}{\varepsilon \hbox{'}}=2 \tan \delta $$

As discussed in the section about microwave properties of plant tissues, the loss tangent of distilled water at 2.4 GHz is 0.12 and 0.3 for an assumed ionic conductivity of 14,000 μS/cm. In other words, within the framework of perturbation theory and the assumption that the losses are due to water and ions only, the anlysis yields a nearly ten times higher conductivity than reported in the literature. In order to resolve this puzzle, we took a closer look at the *IQS* and *FRS* values of Mode 0, because the separation of ionic conductor losses from water dipole relaxation losses is much more pronounced at this low frequency (Table 1). In order to improve the measurement statistics, we measured *FRS* and *IQS* for 6 fresh leaves from one plant (indicated by the numbers in Table 1). Each of the listed *FRS* and *IQS* values corresponds to the average of five subsequent measurements performed on one leaf, the quoted error represents the standard deviation of these five subsequent measurements.

**Table 1:** **Measured FRS and IQS (f = 150 MHz, Mode 0) for 6 different fresh leaves of one wheat plant and calculated ionic conductivity**

**L. no**	***FRS***	**Δ**_**FRS**_ **/** ***FRS*** **[%]**	***IQS***	**Δ**_**IQS**_ **/** ***FRS*** **[%]**	**σ[μS/cm]**	**Δ**_**σ**_ **/σ [%]**
1	1.30⋅10^−5^	40	5.25⋅10^−5^	27	1.4⋅10^4^	48
2	1.47⋅10^−5^	26	6.11⋅10^−5^	15	1.4⋅10^4^	30
3	1.27⋅10^−5^	46	7.78⋅10^−5^	27	2.0⋅10^4^	53
4	1.86⋅10^−5^	21	6.84⋅10^−5^	52	1.2⋅10^4^	56
5	1.94⋅10^−5^	7	8.79⋅10^−5^	20	1.5⋅10^4^	21
6	1.96⋅10^−5^	29	9.52⋅10^−5^	15	1.6⋅10^4^	14
AVE					1.46⋅10^4^	14

Each data point corresponds to the average of 5 subsequent measurements and ionic conductivity σ is determinded from *IQS*/*FRS* by Eq. 6. AVE – weighted average.

The conductivity is proportional to *IQS*/*FRS* (Eqs. 1 and 5)

6 $$ \sigma =\omega {\varepsilon}_0{\varepsilon}_r \tan \delta =\frac{\omega {\varepsilon}_0{\varepsilon}_r}{2}\frac{IQS}{FRS} $$

with ε_r_ ≈ 78 representing the real part of the permittivity of water at the measurement frequency of 150 MHz. The quoted value (1.46 ± 0.20) μS/cm corresponding to the weighted average of the six leaves is in agreement with literature data [21]. To the best of our knowledge, this is the first non-invasive determination of the conductivity of the fluid inside a plant leaf.

As a possible explanation for the enhanced loss tangent measured at 2.4 GHz, it is likely that higher dielectric relaxation losses than assumed for free water may occur due to a high abundance of surface water, which has a significantly higher loss tangent than bulk water at 2.4 GHz [25,26]. The observed slight increase of *IQS*/*FRS* at 2.4 GHz with increasing weight loss is likely due to an increase of the ratio of surface to bulk water as result of faster evaporation of bulk water. In fact, the relatively small variation is far below the expectation of 50% water loss by evaporation, which is supportive for the hypothesis that surface water may contribute to the losses by a significant amount. Comparative measurements with Mode 0 at 150 MHz of sufficient accuracy and other frequencies may help to resolve this puzzle in the future.

**Figure 4:**
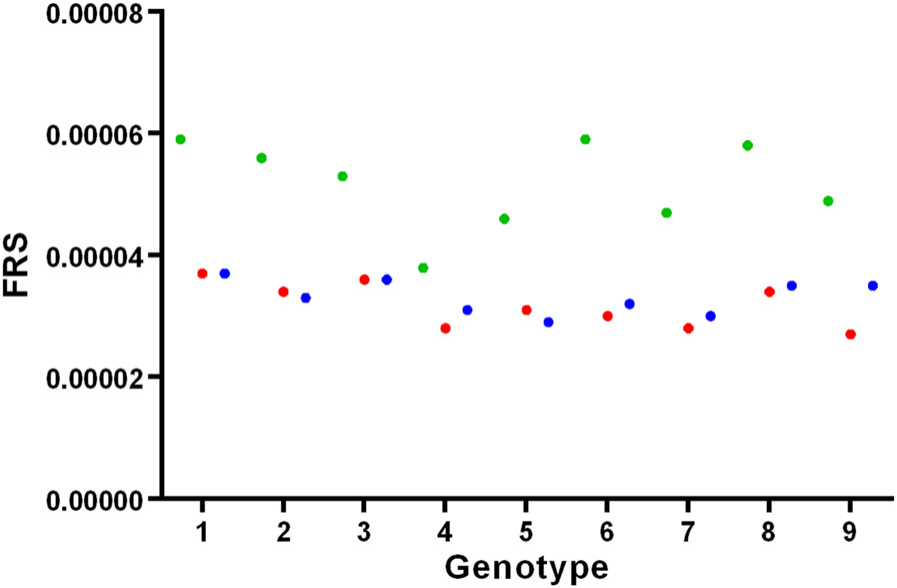
**Analysis of wheat leaves from nine genotypes after 15 days of salt stress.** Green dots represent control, red and blue dot leaves stressed with 100 mM NaCl and 50 mM Na_2_SO_4,_ respectively. X-axes represent the genotypes analyzed (numbered 1–9) and the y-axes the corresponding FRS values.

Furthermore, in order to demonstrate the practical applicability of our microwave technique, wheat plants being challenged by salt stress were measured (at the moment only by Mode 1). The measured *FRS* values reveal a clear difference between the control leaves and the stressed ones (Figure 4). The decrease in the *FRS* value is likely to be linked to an increase of osmolarity induced by salt stress which is adversely affecting the uptake of water by the roots [27,28].

**Figure 5:**
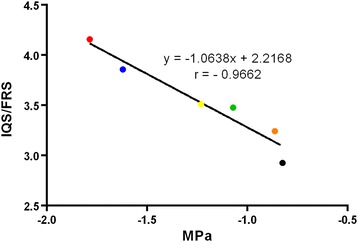
**Correlation between osmotic potential and IQS/FRS values for canola leaves.** Dot-colors indicate measurement of leaves with water content decreasing stepwise: black – 100% (initial fresh weight); brown – 90%, green – 80%; yellow – 70%; blue – 60% and red – 50% of the initial weight. Each dot represents the mean values from four leaves. For each leave, two measurements were performed to define osmotic potential and five for the *IQS*/*FRS* value.

A reduction of the water content in the plant cell leads to an increase of osmolarity. Therefore, the osmotic potential of canola leaves at 6 time-points was determined. A strong negative correlation (r = − 0.97) between *IQS*/*FRS* values and the respective osmotic potential of the leaves at different steps of water reduction was found (Figure 5).

## Material and methods

### Plant material and growth conditions

Four species belonging to different classes of plant kingdom were selected: wheat (*Triticum aestivum* L.) cultivar Zentos, maize (*Zea mays* L.) cultivar Aurelia, potato (*Solanum tuberosum* L.) cultivar Linda and canola (*Brassica napus* L.), cultivar Expert. The plants were grown under greenhouse conditions in pots filled with soil (clay peat mix) and watered regularly.

For the salt stress experiment, nine wheat genotypes were grown in three replicates in aerated hydroponic system (unpublished data). The tested wheat genotypes were Zentos, Syn086 and 7 progenies of the cross between Zentos and Syn086 [29] which were selected based on their performance under salinity stress, representing salt tolerant and salt sensitive genotypes.

The stress was induced by adding to the nutritional solution either NaCl or Na_2_SO_4,_ to end-concentration of 100 mM and 50 mM, respectively.

EC at control = 2.5 mS, NaCl = 11.5 mS, Na_2_SO_4_ = 9.5 mS), pH was checked every day and adjusted at 6.1 to 6.4. The stress was induced at three leave developmental stage (BBCH 13) and lasted for 15 days.

### Measurements of salt stressed plants using the microwave cavity technique

**Figure 6:**
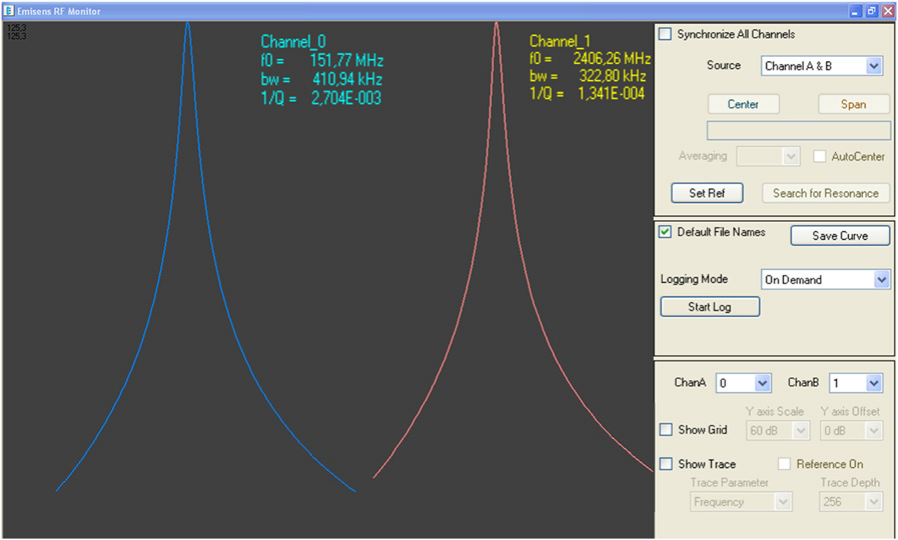
**Screenshot of the user interface of the dual-mode sensor system.** Blue and red curves display the measured resonance of a measurement of modes 0 and 1, respectively. Shown are the values of resonance frequency (f0), inverse Q factor and 3 dB bandwidth bw = f_0/Q for both modes, as determined by a Lorentz fit (Eq. 2). The axis of these plots (horizontal = frequency), (vertical = detector voltage) are not depicted on the screenshot, the control panel on the right hand side of the screen shot is not relevant for the presented analysis.

The measurements were performed using a prototype of EMISENS’s dual-mode sensor system, which was purpose-designed for this study. The quantities *IQS* and *FRS* were determined from the resonant frequency and *Q* factor, as determined by a fit of a Lorentzian to the measured resonant curves displayed in Figure 6. The leaves were pressed by a transparent plastic cover against the aperture of the dual-mode cavity. In case of small leaves which do not fully cover the aperture the position was optimized for maximum *FRS*, which corresponds to the alignment of an elongated leaf (like the one depicted in in Figure 1) perpendicular to the radial metallic rod at a distance from the center corresponding to about half the radius of the dielectric resonator.

For measurements on different leaves of one plant species care was taken to ensure that nearly identical measurement positions were used. The plants were removed from the hydroponic boxes and one leaf of them was placed on the window of the sensor (Figure 1). Five measurements were performed for each leave without changing the position (technical replicates). Immediately after, the undamaged plants were returned into the hydroponic vessels.

### Measurements of water content

In order to follow the kinetics of water content the measurements were performed on detached leaves from the corresponding plants.

The stepwise reduction of water content in leaves was achieved by incubating them at high temperatures. The gravimetric measurement of water loss in the leaves was done by weighting them before and after drying. Shortly, after removal from the plant the leaves were weighted and measured with the microwave sensor system. This first time point was considered as reference for a leaf with 100% (w/w) water. After that, the leaves were placed in an incubator at 45°C until 10% of initial water content was lost and the microwave assessment was performed instantaneously. The drying procedure with 10% loss each step and subsequent microwave measurement was repeated 5 times until reduction to 50% of the initial weight.

### Measurement of the osmotic potential

Leaves of canola plants were detached and after the microwave measurements they were analyzed with respect to their osmotic potential. This was repeated for each step of water reduction as described above. The sap of the leaves was extracted by squeezing them using a garlic presser. Fifteen μl sap-solution was employed to define the osmotic potential using an Osmomat (Osmomat 030-D, Gonotec GmbH, Berlin, Germany). The conversion of the osmolality values (osmol/kg) in osmotic potential (MPa) as described by Pariyar, et al. [30].

## Conclusions

We have demonstrated non-invasive assessment of the water content by an evanescent field microwave sensor at 2.4 GHz for four different species of plant leaves due to a comparative study with gravimetric data. Our approach was proven to be highly reproducible and applicable for leaves of various size, shape and thickness. The frequency shift versus water content curves are slightly sub linear for the larger leaves, which may result from the inhomogeneous water distribution in the veins. For canola leaves, a strong correlation between the measured ratio of loss and frequency shift data to the osmotic potential was found, which indicates that the microwave method can be used for contact-free assessment of the osmolytes status of a plant. Due to the combination of a microwave (*f* = 2.5 GHz) and a sub-microwave frequency (*f* = 150 MHz) in one sensor device the method has a strong potential for simultaneous non-invasive assessment of water and salt status in a single leaf under test.

For the future, a down-scaled system operated at higher frequencies may be developed in order to achieve a higher reproducibility for the assessment of smaller leaves. The optimization of the design of the dual mode sensor and a further refinement of the electronic modules and the employed algorithm for accurate measurements of small changes of the resonant parameters should enable the simultaneous study of water content and average mineral content.

We expect that our technique may advance to a standard tool for hydration monitoring in plants in the near future. A lightweight portable version for assessment of plants in the field is currently under development. This may enable the realization of knowledge-based watering systems as integral procedure of precision agriculture in the future.
